# Electrochemical sensor based on a three dimensional nanostructured MoS_2_ nanosphere-PANI/reduced graphene oxide composite for simultaneous detection of ascorbic acid, dopamine, and uric acid†

**DOI:** 10.1039/c8ra09511f

**Published:** 2019-01-23

**Authors:** Shuaihui Li, Yashen Ma, Yongkang Liu, Gu Xin, Minghua Wang, Zhihong Zhang, Zhongyi Liu

**Affiliations:** School of Chemical Engineering and Energy, Zhengzhou University No. 100 Science Avenue Zhengzhou 450001 Henan China liuzhongyi@zzu.edu.cn 2006025@zzuli.edu.cn +86-37186609676; Henan Provincial Key Laboratory of Surface & Interface Science, Zhengzhou University of Light Industry Zhengzhou 450002 China; College of Chemistry and Molecular Engineering, Zhengzhou University Zhengzhou 450001 China guxin@zzu.edu.cn

## Abstract

A three dimensional (3D) nanostructured composite based on the self-assembly of MoS_2_ nanospheres and polyaniline (PANI) loaded on reduced graphene oxide (denoted by 3D MoS_2_-PANI/rGO) was prepared *via* a feasible one-pot hydrothermal process. The 3D MoS_2_-PANI/rGO nanocomposite not only exhibits good functionality and bioaffinity but also displays high electrochemical catalytic activity. As such, the developed 3D MoS_2_-PANI/rGO nanocomposite can be employed as the sensing platform for simultaneously detecting small biomolecules, *i.e.*, ascorbic acid (AA), dopamine (DA), and uric acid (UA). The peak currents obtained from the differential pulse voltammetry (DPV) measurements depended linearly on the concentrations in the wide range from 50 μM to 8.0 mM, 5.0 to 500 μM, and 1.0 to 500 μM, giving low detection limits of 22.20, 0.70, and 0.36 μM for AA, DA, and UA, respectively. Furthermore, the 3D MoS_2_-PANI/rGO-based electrochemical sensor also exhibited high selectivity, good reproducibility and stability toward small molecule detection. The present sensing strategy based on 3D MoS_2_-PANI/rGO suggests a good reliability in the trace determination of electroactive biomolecules.

## Abstract 2

A three dimensional (3D) nanostructured composite based on the self-assembly of MoS_2_ nanospheres and polyaniline (PANI) loaded on reduced graphene oxide (denoted by 3D MoS_2_-PANI/rGO) was prepared *via* a feasible one-pot hydrothermal process.

## Graphical abstract

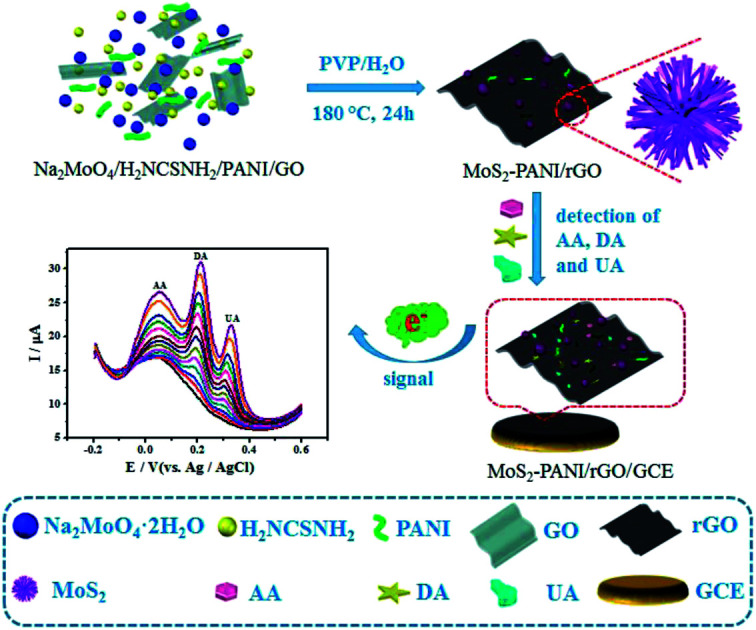

## Introduction

Ascorbic acid (AA), dopamine (DA), and uric acid (UA) are crucial biomolecules that usually coexist in human physiological fluids.^1–3^ Abnormal DA level in the central nervous system may lead to several neurological diseases, *e.g.*, schizophrenia and Parkinson's disease.^4,5^ Similarly, AA associates to atherosclerosis,^6^ and abnormality of UA level in the human metabolism causes diseases like hyperuricemia and gout.^7^ Therefore, sensitive and rapid detection of AA, DA, and UA in biological systems is vital for healthcare, routine analysis and clinical diagnostics. Several analytical techniques including chromatography,^8^ surface plasmon resonance spectroscopy,^9^ chemiluminescence,^10^ fluorescence,^11^ and electrochemical analysis,^12^ have been conducted for detecting biomolecules. Among them, electrochemical techniques have been receiving considerable interest because of the low cost, fast response, easy operation, and inexpensive instrumentation required.^13,14^ Notably, as electroactive molecules, AA, DA, and UA can be detected simultaneously by using electrochemical technique.^15^ However, the electro-catalytic oxidation of these biomolecules at bare electrodes occur at very similar potentials, leading to poor selectivity.^16^

Many efforts have been made to develop effective electrochemically active and large surface area sensors with highly selective and sensitive electrochemical current responses. To address these issues, various sensing materials, *e.g.*, metal nanoparticles, semiconductors, polymers and carbon materials, have been employed.^17–19^ With high electron transfer rate, electrochemical active surface area, and chemical functionality, reduce graphene oxide (rGO) has been investigated widely in electrochemical sensing.^20,21^ Chen *et al.* reported that cobalt manganese oxides increased the amount of graphene captured in the composites and improved sensing activities.^22,23^ Moreover, as a two-dimensional (2D) layered nanomaterial, molybdenum disulfide (MoS_2_) has been focused on the fields like energy conversion and storage, electronic devices, catalysts, and sensors,^24–28^ due to its highly optical transmittance, unique electrochemical properties, and large electrochemical active surface area. MoS_2_-based nanocomposites have been used as matrix for simultaneously detecting of AA, DA, and UA. For instance, the gold nanoparticle-decorated MoS_2_ nanocomposite modified electrode exhibited good electro-catalytic oxidation of AA, DA, and UA, giving detection limits of 100, 0.05, and 10 μM, respectively.^29^ Interestingly, MoS_2_/graphene hierarchical frameworks have been reported where Mo^6+^ cations were found to induce the self-assembly of graphene oxide, and the aerogel structures depend strongly on different MoS_2_ loadings.^30^ What's more, most of the reported MoS_2_ used as the sensing materials for biomolecules detection were still based on 2D planar structures. Considering that the effective electrochemically active surface areas of MoS_2_ for adsorption and electro-catalytic oxidation of biomolecules may be closely associated with their morphology, the structure modulation needs to be further studied.^31,32^ Diverse shapes of nanoparticles distributed and anchored on graphene sheets can be achieved by varying synthesis conditions.^33,34^ Additionally, conductive polymers such as polyaniline (PANI) have also been used to functionalize MoS_2_ to improve the electro conductivity of the materials for the detection of dopamine.^35^ MoS_2_/PANI/rGO aerogel nanocomposite exhibited excellent long-term cycling stability and a high capacity.^36^ RGO/MoS_2_/PANI@AuNPs nanocomposite-based electrochemical aptasensor was also reported for detection of aflatoxin B1.^37^ However, the nanocomposites were synthesized *via* multiple steps.

**Scheme 1:**
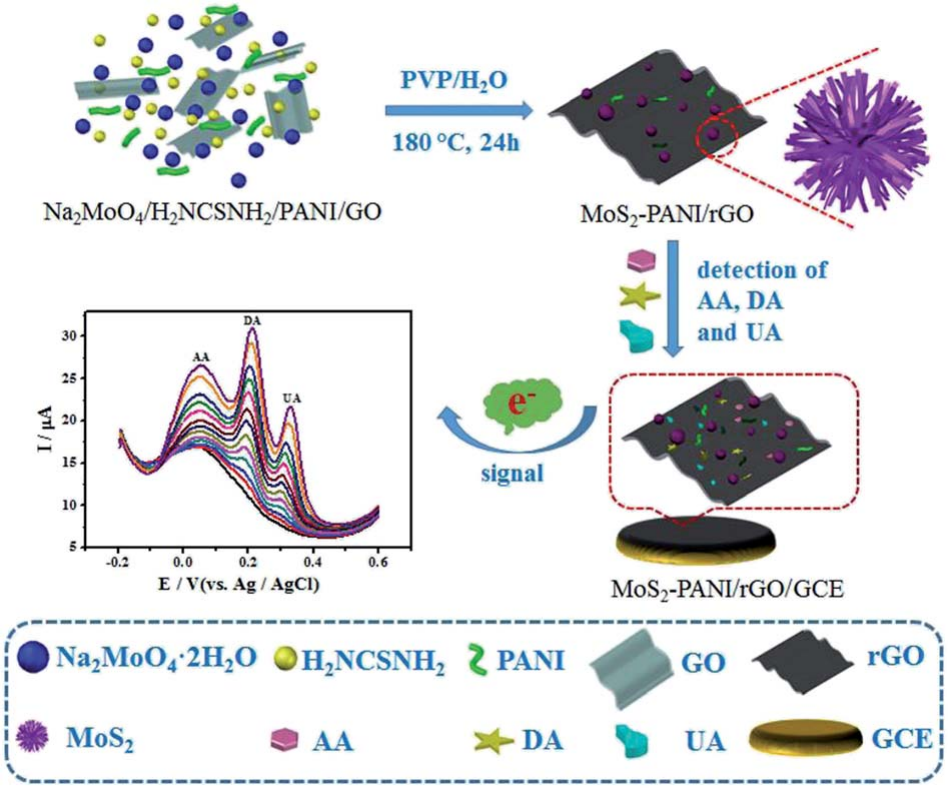
Schematic diagram of MoS_2_-PANI/rGO-based electrochemical biosensor for simultaneous detection of AA, DA, and UA.

This work aims to enhance the electrochemical activity of the MoS_2_/graphene and improve the bioaffinity between the small biomolecules and the sensing layer. 3D nanostructured composite based on the self-assembly of MoS_2_ nanospheres and PANI loaded on rGO (3D MoS_2_-PANI/rGO) was synthesized by a one-pot hydrothermal reaction, following by the application as electrochemical catalyst toward the trace determination of biomolecules in blood and urine samples (Scheme 1). As expected, the synergistic effects of the effective electro-catalytic performance of MoS_2_, electrochemically activity of PANI, and the excellent charge transfer of graphene impart superior electro-catalytic activity of 3D nanostructured MoS_2_-PANI/rGO composite for the oxidation reactions of AA, DA, and UA.

## Experimental

### Materials and chemicals

Graphite powders, potassium permanganate (KMnO_4_), sulfuric acid (H_2_SO_4_), 30% H_2_O_2_ solution, sodium molybdate (Na_2_MoO_4_·2H_2_O), thiourea (H_2_NCSNH_2_), polyvinylpyrrolidone (PVP), aniline, ammonium persulfate, HClO_4_, AA, DA, and UA were supplied by Aladdin reagent (Shanghai, China) and used as received. Milli-Q water (≥18.2 MΩ cm) was used throughout the experiments. The 0.1 M phosphate buffer solution (PBS, pH 7.0) was prepared by mixing stock solutions of Na_2_HPO_4_ and KH_2_PO_4_.

### Preparation of 3D MoS_2_-PANI/rGO nanocomposite

The preparation of GO and PANI are described in detail in the ESI (S1).† MoS_2_-PANI/rGO nanocomposite was synthesized by a one-pot hydrothermal approach. Briefly, 0.40 g Na_2_MoO_4_·2H_2_O, 0.66 g thiourea, 0.20 g PANI and 0.25 g PVP were added in 40 mL of GO aqueous dispersion (1.25 mg mL^−1^) to form a homogeneous solution under stirring. Then the solution was transferred into a 50 mL Teflon-lined stainless steel autoclave and heated at 180 °C for 24 h. After cooling to ambient temperature, the precipitate was collected by centrifugation and washed thoroughly using ultrapure water and ethanol. Finally, the solid was dried in a vacuum oven at 60 °C for 12 h and the 3D MoS_2_-PANI/rGO nanocomposite was then obtained. MoS_2_/rGO composite was prepared in a similar manner in the absence of PANI.

### Apparatus

X-ray diffraction (XRD) spectra were performed using a D8 advance X-ray diffraction instrument (Germany) in the 2*θ* range of 5–80°. Fourier-transform infrared (FT-IR) spectra were recorded on a Bruker TENSOR27 instrument from 400 to 4000 cm^−1^. The X-ray photoelectron spectroscopy (XPS) analysis was measured by a Thermo Fisher ESCALAB 250Xi spectrometer equipped with an Al anode (Al-Kα 1486.6 eV). Surface morphologies of the samples were investigated by field-emission scanning electron microscopy (FE-SEM, JSM-6490LV, Japan) and transmission electron microscopy (TEM, JEM-2100F, Japan), respectively.

### Fabrication of biosensor and electrochemical measurements

The glassy carbon electrodes (GCE, 3.0 mm in diameter) were polished using 0.3 μm and 0.05 μm alumina slurry, followed by sonicating in nitric acid, ethanol, and ultrapure water sequentially, and drying at room temperature. Afterward, 10 μL of the MoS_2_-PANI/rGO suspension (1.0 mg mL^−1^) was dropped onto GCE electrode surface, then dried in the ambient air to develop the sensor (MoS_2_-PANI/rGO/GCE). For comparison, the MoS_2_/rGO/GCE and PANI/GCE were made in the same way.

Electrochemical measurements were performed on a CHI660D electrochemical workstation (Chenhua, Shanghai, China) with a conventional three-electrode system. The GCE or modified GCEs, platinum slide, and Ag/AgCl (saturated KCl) electrode were used as working electrode, counter electrode, and reference electrode, respectively. Cyclic voltammetry (CV) was recorded in 0.1 M PBS (pH 7.0; containing 0.1 M KCl). The potential range was from −0.2 to 0.8 V and the scan rate was 50 mV s^−1^. Differential pulse voltammetry (DPV) was collected at from −0.2 V to 0.8 V with pulse amplitude of 50 mV.

## Results and discussion

### Characterization

**Fig. 1:**
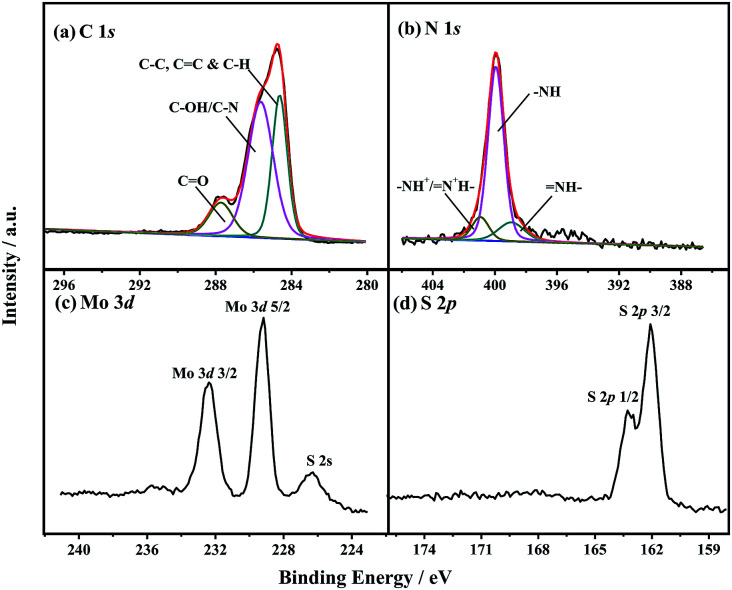
High-resolution XPS spectra of (a) C 1s, (b) N 1s, (c) Mo 3d, and (d) S 2p of MoS_2_-PANI/rGO nanocomposite.

The chemical structure and components of nanomaterials were determined by FT-IR (S2†) and XPS. The FT-IR spectra (Fig. S1†) suggested that PANI was present in MOS_2_-PANI/rGO nanocomposite. The chemical composition and valence states of C, N, Mo and S containing in MoS_2_/rGO and MoS_2_-PANI/rGO (Fig. 1, S2 and Table S1†) nanocomposites were investigated by XPS in detail. From the XPS survey spectrum (Fig. S2a†), C, N, O, Mo and S can be clearly identified in MoS_2_-PANI/rGO with atomic% of 74.5, 11.8, 10.8, 0.9, and 2.0, respectively. Fig. 1a shows the C 1s characteristic peak which is caused by the component of rGO and PANI. Three main peaks are centered at 284.6, 285.6, and 287.7 eV, which are assigned with the groups of C–C/C=C/C–H, C–OH/C–N, and C=O, respectively.^38^ The graphene possess numerous edge planes and defects, which are conductive to the fast electron transfer as well as electro-catalytic activity.^39,40^ Apparently, the C–N was originated from PANI. The N 1s core-level XPS spectrum (Fig. 1b) was fitted into three components, 398.8, 399.8 and 400.8 eV, which are corresponded to imine (=N–), amine (–NH), and protonated amine units (–NH^+^/=N^+^–) containing in PANI, respectively.^41^ The amine and imine groups can form hydrogen bonds with hydroxyl groups present in the small biomolecules and eventually results strong bioaffinity.^42^Fig. 1c and d depict the high-resolution scan XPS spectra of the Mo 3d, S 2s and S 2p electrons. The binding energies of Mo 3d_3/2_, Mo 3d_5/2_ and S 2s peaks are located at 232.4, 229.2 and 226.5 eV, whereas S 2p_1/2_ and S 2p_3/2_ peaks are observed at 163.3, and 162.1 eV, respectively,^30,43^ suggesting that Mo^4+^ chemical state existed and the formation of crystalline MoS_2_. The combination of the MoS_2_, rGO and PANI may effectively increase the conductivity, bioaffinity and catalytic activity of the nanocomposite.

### Morphological studies

**Fig. 2:**
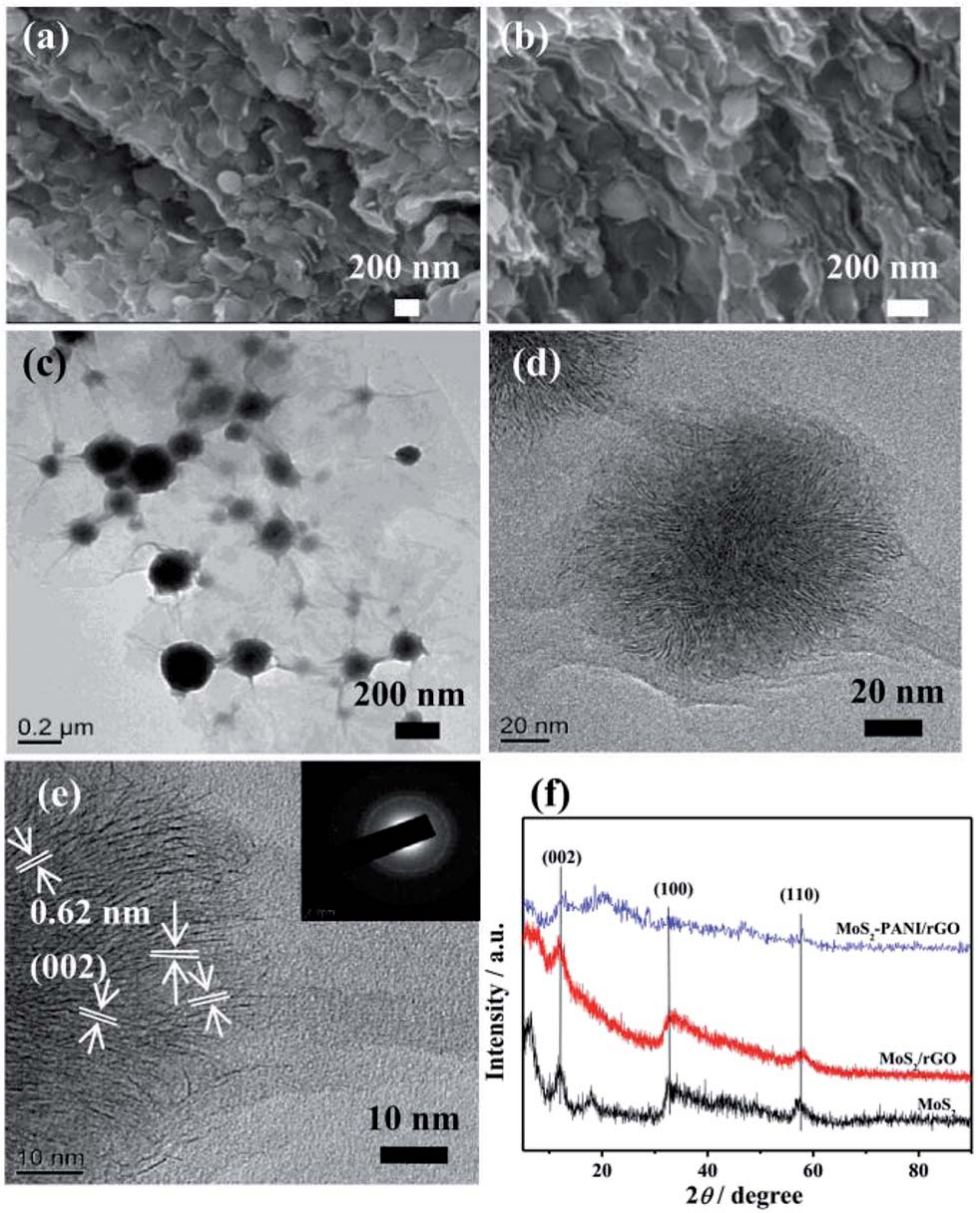
(a and b) FE-SEM images of MoS_2_-PANI/rGO nanocomposite; (c) TEM and (d and e) HR-TEM images of 3D MoS_2_-PANI/rGO nanocomposite (the inset in (e) is the corresponding SAED pattern), and (f) XRD patterns of the MoS_2_, MoS_2_/rGO and MoS_2_-PANI/rGO samples.

The surface morphology of as-prepared MoS_2_/rGO, PANI, and MoS_2_-PANI/rGO were investigated by FE-SEM, as shown in Fig. 2 and S3.† The results show that the MoS_2_ nanospheres were formed with a diameter of 300–400 nm (Fig. S3a†), approximately, whereas the PANI exhibits a rod-like shape (Fig. S3b†). The FE-SEM images of MoS_2_/rGO reveal spherical-shaped MoS_2_ nanoparticles wrapped by crumpled structure of graphene sheets (Fig. S3c and d†). As for the MoS_2_-PANI/rGO, the honeycomb-like structure was formed with MoS_2_ nanospheres interpenetrating in 3D graphene sheets (Fig. 2a and b), which could increase the electrochemical active surface area of the nanocomposite. Further structural insights were obtained in TEM analysis. The graphene appears a curved and crinkled texture (Fig. 2c, d and S4a–c†), which is well anchored by MoS_2_ nanospheres. For MoS_2_-PANI/rGO, dendritic hierarchical structure with several layers can be found in high-resolution TEM (HR-TEM) image. In Fig. 2e, the layered structures with an interlayer distance of ∼0.62 nm corresponds to the (002) crystal planes of MoS_2_. The corresponding selected area electron diffraction (SAED) patterns of MoS_2_/rGO (Fig. S4d†) and MoS_2_-PANI/rGO (inset of Fig. 2e) confirm the hexagonal structure of MoS_2_ with different crystallographic orientations and present separated diffraction rings that can be indexed to the XRD patterns (Fig. 2f). The peaks that appeared in the XRD patterns of all the three samples are indexed based on MoS_2_.

The major diffraction peaks at 2*θ* = 14.4°, 32.7°, and 58.3° are in accordance with the (002), (100), and (110) crystal planes of hexagonal MoS_2_ (JCPDS 37-1492).^44,45^ Nevertheless, the intensity of diffraction peak for MoS2-PANI/rGO decreased, indicating that PANI slightly affected the crystallization performance of MoS_2_.

### Electrochemical performance of the as-synthesized nanomaterials

**Fig. 3:**
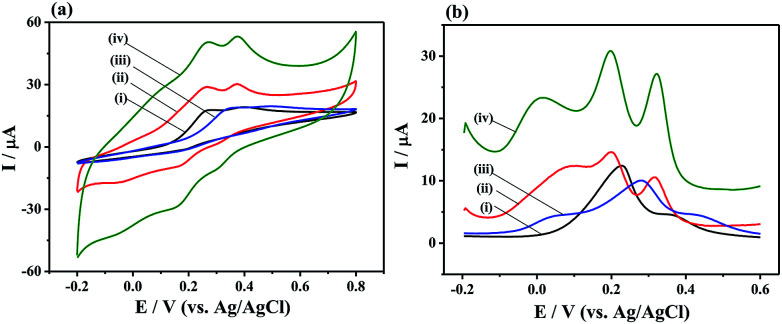
(a) CV and (b) DPV curves of (i) bare GCE, (ii) MoS_2_/rGO/GCE, (iii) PANI/GCE, and (iv) MoS_2_-PANI/rGO/GCE in 0.1 M PBS (pH 7.0, 0.1 M KCl) containing 2.0 mM AA, 150 μM DA, and 200 μM UA.

To evaluate the electrochemical performance of the different materials, CV curves were tested on the bare GCE, MoS_2_/rGO, PANI, and 3D MoS_2_-PANI/rGO-modified glassy carbon electrodes. The MoS_2_-PANI/rGO/GCE (0.125 cm^2^) exhibited larger electrochemical active surface area than MoS_2_/rGO/GCE (0.101 cm^2^) and PANI/GCE (0.102 cm^2^), suggesting it can provide quick mass transport of molecules to the electrocatalyst (Fig. S5†). When the AA, DA and UA biomolecules were added in 0.1 M PBS solution (Fig. 3a), the GCE and PANI/GCE showed broad peak from −0.2 to 0.8 V, while MoS_2_/rGO/GCE had two obvious peak responses to DA and UA. In comparison, the MoS_2_-PANI/rGO/GCE exhibited the largest anodic peaks in the CV measurements. Three peaks were observed on MoS_2_-PANI/rGO/GCE in DPV measurements, indicating a simultaneous electrochemical detection of the three bio-molecules (Fig. 3b). The MoS_2_-PANI/rGO/GCE exhibits the highest peak current intensity and three well-resolved peaks at 0.020, 0.196, and 0.320 V (Δ*E*_AA–DA_ = 176 mV, Δ*E*_DA–UA_ = 124 mV, Δ*E*_AA–UA_ = 300 mV), implying that synergistic effect existed among the MoS_2_, rGO and PANI toward the electrochemical catalysis of three small biomolecules. The observed anodic peaks are ascribed to the oxidation of hydroxyl groups to carbonyl groups in AA, catechol to *o*-quinone in DA, and bridging double bond to hydroxyl groups in UA.^16^

**Fig. 4:**
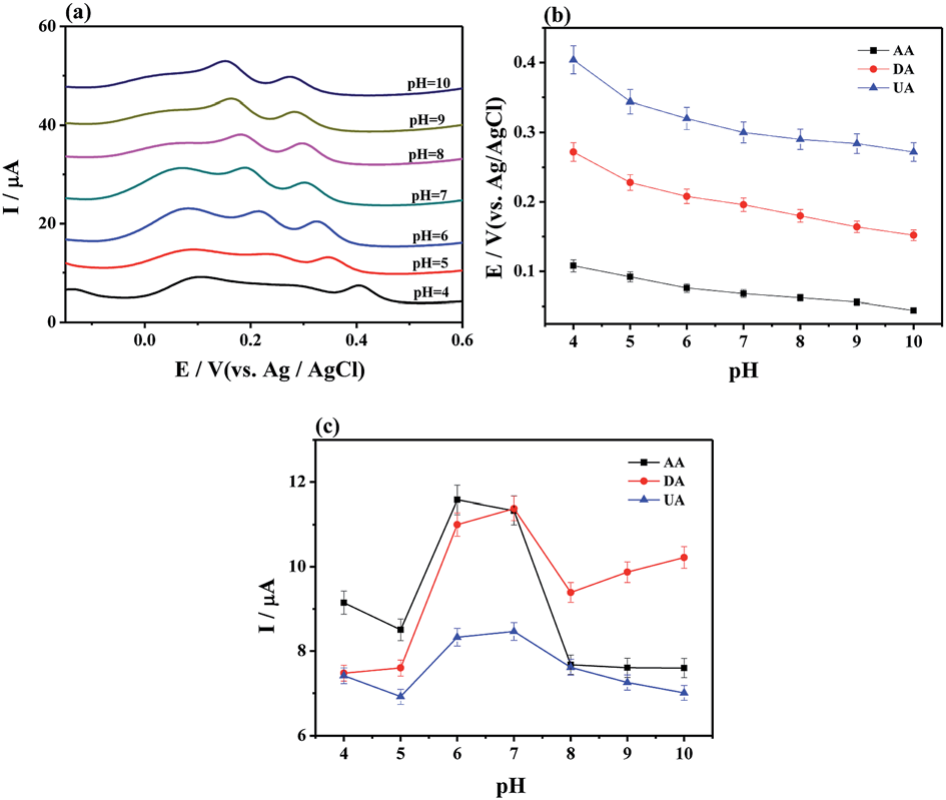
(a) DPVs of MoS_2_-PANI/rGO/GCE in 0.1 M PBS (pH 7.0, 0.1 M KCl) containing 1.0 mM AA, 75 μM DA, and 75 μM UA with various pH value with in the range from 4.0 to 10.0, and the corresponding peak potential (b) and peak current (c) *versus* the pH values.

Moreover, the effect of pH on electrochemical oxidation of AA, DA, and UA with MoS_2_-PANI/rGO/GCE was investigated (Fig. 4). The results show that the anodic peak potential of AA, DA, and UA shifted negatively with increasing of their pH value (pH 4.0–10.0) (Fig. 4a), and oxidation peak potential of AA, DA, and UA were linearly proportional to the pH value (Fig. 4b), demonstrating that the proton took part in the electrochemical oxidation reaction process.^6,29^ The peak current of AA, DA, and UA was also changed with the pH value. The AA, DA, and UA achieved the maximum value at pH 6.0, 7.0, and 7.0, respectively (Fig. 4c). Considering sensitivity and selectivity, the electrolyte solution with pH 7.0 was selected for further measurements of the analytes.

### Simultaneous detection of AA, DA and UA

**Fig. 5:**
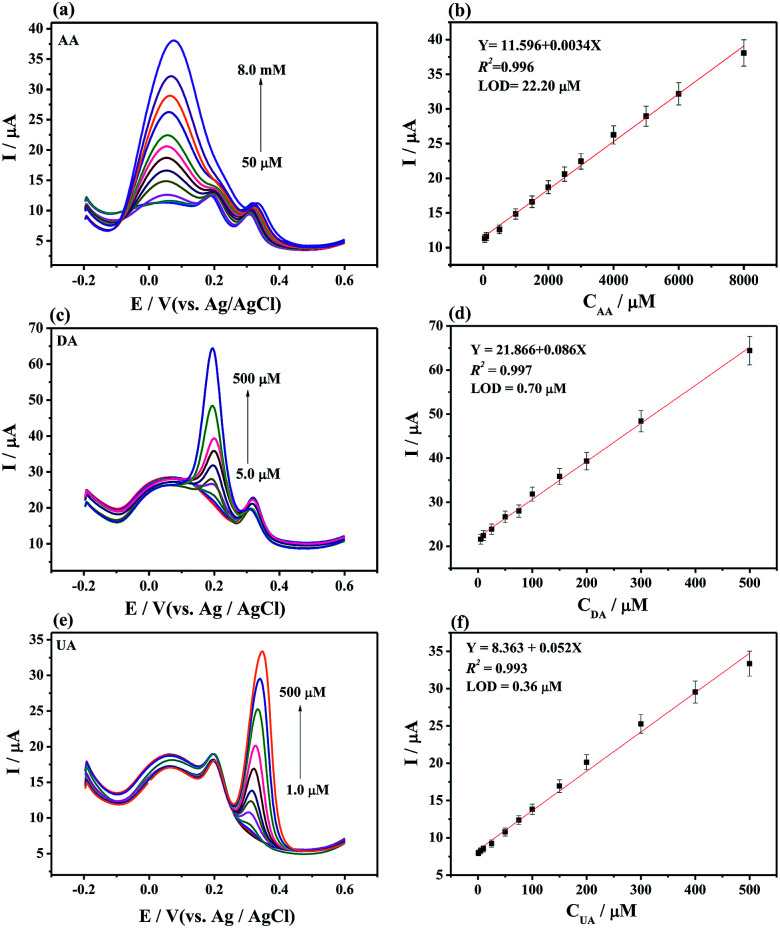
(a) DPVs of MoS_2_-PANI/rGO/GCE and (b) the plot of peak currents *vs.* concentrations of AA from 50 μM to 8.0 mM with 75 μM DA and 75 μM UA. (c) DPVs of MoS_2_-PANI/rGO/GCE and (d) the plot of peak currents *vs.* concentrations of DA from 5.0 to 500 μM with 1.0 mM AA and 75 μM UA. (e) DPVs of MoS_2_-PANI/rGO/GCE and (f) the plot of peak currents *vs.* concentrations of UA from 1.0 μM to 500 μM with 1.0 mM AA and 75 μM DA. The solution was PBS (pH 7.0, 0.1 M) containing 0.1 M KCl in the experiment.

Individually or simultaneously analyze AA, DA, and UA on MoS_2_-PANI/rGO/GCE was studied by DPV. It is found that the response current corresponding to the analyte increases with increasing concentration of the ternary mixture. In Fig. 5a–b, the peak currents increased linear with the AA concentrations in the range of 50 μM to 8.0 mM. The regression equation of *I*_p,AA_ (μA) = 11.596 + 0.0034*C*_AA_ (μM) (*R*^2^ = 0.996) was obtained. The limit of detection (LOD) was determined to be 22.20 μM for AA at a signal-to-noise ratio of 3. Similarly, the peak currents of DA (Fig. 5c and d) and UA (Fig. 5e and f) were proportional to the concentration in the range of 5.0–500 μM (DA) and 1.0–500 μM (UA). The calibration equations were *I*_p,DA_ (μA) = 21.866 + 0.086*C*_DA_ (μM) (*R*^2^ = 0.997) and *I*_p,UA_ (μA) = 8.363 + 0.052*C*_UA_ (μM) (*R*^2^ = 0.993) with LOD of 0.70 μM for DA and 0.36 μM for UA, respectively.

**Fig. 6:**
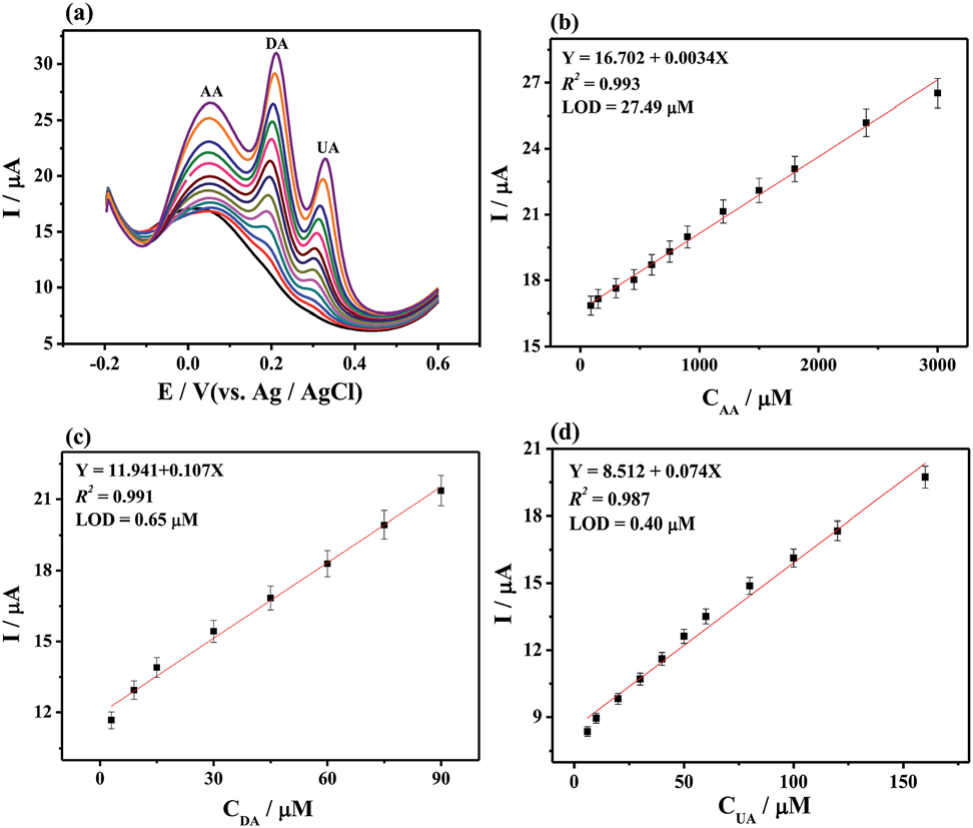
(a) DPV curves of MoS_2_-PANI/rGO/GCE in 0.1 M PBS (pH 7.0, 0.1 M KCl) containing AA, DA, and UA with different concentrations. The concentration from bottom to top: 30 μM to 3.0 mM for AA, 3.0 to 300 μM for DA, and 2.0 to 200 μM for UA, respectively. The plots of the oxidative peak currents *versus* the concentrations of (b) AA, (c) DA, and (d) UA.

In the simultaneous detection of AA, DA, and UA experiments, three well- resolved anodic peaks at potentials of 0.052, 0.196, and 0.304 mV were presented at MoS_2_-PANI/rGO/GCE from DPV plots (Fig. 6). The three peak currents were linearly dependent on the concentrations ranging from 30 μM to 3.0 mM for AA, 3.0 to 300 μM for DA, and 2.0 to 200μM for UA while the LOD of AA, DA, and UA were 27.49, 0.65, and 0.40 μM, respectively. The results demonstrate that the 3D MoS_2_-PANI/rGO-based sensor exhibited wide linear ranges and low detection limits for detection of biomolecules comparative to those of other sensors (Table 1).

**Table 1:** Comparison of sensors for detection of AA, DA, and UA

Electrode	Method	Linear range (μM)			LOD (μM)			Reference
		AA	DA	UA	AA	DA	UA	
RGO-ZnO/GCE	DPV	50–2350	1–70	3–330	3.71	0.33	1.08	6
AuNPs@MoS_2_/GCE	DPV	1000–70 000	0.05–4000	10–7000	100	0.05	10	29
Fe-Meso-PANI/GCE	LSV^a^	10–300	10–300	10–300	6.5	9.8	5.3	16
GO-PANI/GCE	DPV	150–1050	1–14	3–26	50	0.5	1	46
Au/RGO/GCE	DPV	240–1500	6.8–41	8.8–53	51	1.4	1.8	47
Graphene/SnO_2_/GCE	DPV	100–1000	1–20	3–21	100	1	3	48
MoS_2_PANI/rGO/GCE	DPV	50–8000	5.0–500	1.0–500	22.20	0.70	0.36	This work

a LSV is linear sweep voltammetry.

### Selectivity, reproducibility, and stability of the fabricated biosensor

**Fig. 7:**
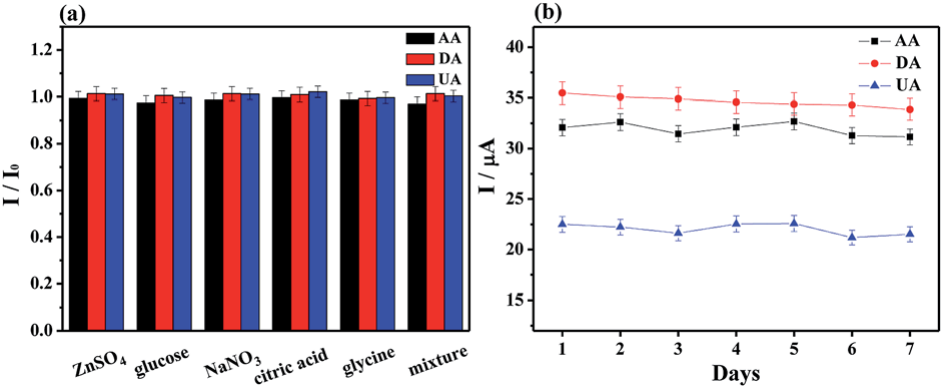
(a) Peak current ratio of MoS_2_-PANI/rGO/GCE to 1.0 mM AA, 75 μM DA, and 75 μM UA in the presence of 1 mM of various interfering substances (ZnSO_4_, glucose, NaNO_3_, citric acid, glycine, and the mixture, respectively). (b) The stability of MoS_2_-PANI/rGO/GCE in 0.1 M PBS (pH 7.0, 0.1 M KCl) containing 1.0 mM AA, 75 μM DA, and 75 μM UA.

Several potential interferential species were used to examine the selectivity at MoS_2_-PANI/rGO/GCE for simultaneously detecting of AA (1.0 μM), DA (75 μM) and UA (75 μM) (Fig. 7a).

There were negligible interferences detected in the presence of NaNO_3_, ZnSO_4_, glucose, glycine, and citric acid, whose concentrations are 1.0 mM. The peak response changes were less than ±3%, revealing a good selectivity of the developed electrochemical sensor.

Five MoS_2_-PANI/rGO modified GCEs were prepared independently and their peak currents for AA, DA and UA mixture were investigated (Fig. S6†). The relative standard deviation (RSD) were 4.0% for AA, 4.56% for DA, and 4.24% for UA, indicating the good reproducibility of the developed sensor. In successive 15 times DPV measurements, the RSD values of 1.13%, 1.70%, and 2.49% for AA, DA and UA were observed. Furthermore, the stability of the electrode was also determined by DPV measurements for 7 days. The oxidation currents decreased by less than 5.0% of the initial currents (Fig. 7b), confirming a good stability of the biosensor.

### Real sample analysis

To evaluate the applicability of the MoS_2_-PANI/rGO-based sensor, the simultaneous detection of AA, DA, and UA in human serum and urine samples was investigated by using standard addition method. Human serum (obtained from Beijing Solarbio Science & Technology Co., Ltd.) and human urine (supplied by an adult male student in the laboratory) were filtered through 0.22 μm filter, and diluted 100 times with 0.1 M PBS (pH 7.0) to prepare the real samples. Subsequently, DPV curves were collected before and after spiking with different concentrations of UA, DA and AA in the real samples (Fig. S7†). As shown in Table 2, the recoveries of the three kinds of small biomolecules varied from 99.0% to 103.5% in human serum, whereas they changed from 96.2 to 103.6% in human urine samples. The RSD values were also accurate and precise, indicating the applicability of the developed sensor for detection of these electroactive molecules in real biological system.

**Table 2:** Detection and recovery of AA, DA and UA in real samples (*n* = 3)^a^

Sample no.	Species	Spiking (μM)	Detected (μM)	Recovery (%)	RSD (%)
Human serum^1^	AA	1200	1241.8	103.5	1.71
	DA	120	119.5	99.6	3.38
	UA	120	121.5	101.2	1.20
Human serum^2^	AA	1800	1810.4	100.6	1.03
	DA	180	183.0	101.7	1.89
	UA	180	178.3	99.0	2.21
Human urine^1^	AA	1200	1199.0	99.9	1.34
	DA	120	121.6	101.3	2.30
	UA	120	124.3	103.6	2.45
Human urine^2^	AA	1800	1783.7	99.1	1.35
	DA	180	180.1	100.0	0.76
	UA	180	173.2	96.2	3.12

a The number “1” and “2” samples were prepared separately with different concentrations of AA, DA, and UA.

## Conclusion

A 3D nanostructured MoS_2_-PANI/rGO nanocomposite based on the self-assembly of MoS_2_ nanospheres and PANI loaded on reduced graphene oxide was synthesized by a one-pot hydrothermal reaction, and employed as the platform for electrochemical oxidation of biomolecules. The result showed that synergistic effect existed among MoS_2_, rGO and PANI. The ternary composite exhibited good electrochemical activity, high bioaffinity and strong catalytic effect on the oxidation of biomolecules. The 3D MoS_2_-PANI/rGO-based sensing platform exhibited high sensitivity for simultaneous detection of AA, DA, and UA with three distinguished oxidation peaks (Δ*E*_AA–DA_ = 176 mV, Δ*E*_DA–UA_ = 124 mV, Δ*E*_AA–UA_ = 300 mV) in DPV measurements. The MoS_2_-PANI/rGO/GCE showed excellent responses toward AA, DA, and UA in the linear range of 50 μM to 8.0 mM, 5.0–500 μM, and 1.0–500 μM with low detection limit of 22.20, 0.70, and 0.36 μM (S/N = 3), respectively. The sensor also exhibited good selectivity, reproducibility and stability for simultaneous detection of these biomolecules in human serum and urine samples.

## Ethical conduct of research

The authors state that for investigations involving human subjects, informed consent was obtained from all human subjects.

## Conflicts of interest

There are no conflicts to declare.

## Supplementary Material

RA-009-C8RA09511F-s001
