# Supplementary material for: Electrochemical sensor based on a three dimensional nanostructured MoS2 nanosphere-PANI/reduced graphene oxide composite for simultaneous detection of ascorbic acid, dopamine, and uric acid
Source: RSC Adv. 2019 Jan 23;9(6):2997–3003. doi: 10.1039/c8ra09511f (PMC9060248; doi:10.1039/c8ra09511f)
Supplement: RA-009-C8RA09511F-s001 [file RA-009-C8RA09511F-s001.pdf]

Electronic Supplementary Material (ESI) for RSC Advances.
This journal is © The Royal Society of Chemistry 2019

Supplementary Information for

Electrochemical sensor base on three dimensional nanostructured MoS_2 nanospheres-PANI/reduced graphene oxide composite for simultaneous detection of ascorbic acid, dopamine, and uric acid

Shuaihui Li[a], Yashen Ma[b], Yongkang Liu[b], Gu Xin[c*], Minghua Wang[b], Zhihong Zhang[b*], Zhongyi Liu[a*]

[a]School of Chemical Engineering and Energy, Zhengzhou University, Zhengzhou 450001, China.

[b]Henan Provincial Key Laboratory of Surface & Interface Science; Zhengzhou University of Light Industry, Zhengzhou 450002, China.

[c]College of Chemistry and Molecular Engineering, Zhengzhou University, Zhengzhou, 450000, China.

*Corresponding authors:

E-mail address: guxin@zzu.edu.cn; 2006025@zzuli.edu.cn; liuzhongyi@zzu.edu.cn

Contents

S1. Synthesis of GO, PANI and MoS_2 nanospheres

S2. Chemical structure and components of the samples

S3. SEM and TEM images of the samples

S4. Electrochemical active surface area of the as-synthesized nanomaterials

S5. Reproducibility of the developed biosensor

S6. Real sample analysis

S1. Synthesis of GO, PANI and MoS_2 nanospheres

Graphene oxide (GO) was prepared by acid oxidation of graphite powers according to the modified Hummers' method.[1] Crude GO was dispersed in 200 mL of ultrapure water and exfoliated with a Cell crusher (900-1000 W) for 2 h. After being dried using freeze drier, the GO product was obtained finally. GO (50 mg) was added to 40 mL of ultrapure water and sonicated thoroughly until a homogeneous suspension of GO (1.25 mg mL^{-1}) was obtained. PANI was synthesized according to our previous literature.[2]

MoS_2 nanospheres were prepared by hydrothermal method. Briefly, 0.40 g $Na_2MoO_4 \cdot 2H_2O$, 0.66 g thiourea, and 0.25 g PVP were dissolved in 40 mL of deionized water to form a homogeneous solution. Then the solution was transferred to a 50 mL Teflon-lined stainless steel autoclave and heated in an electric oven at 180 °C for 24 h. After cooling to room temperature, the precipitate was isolated by centrifugation and washed thoroughly with deionized water and ethanol for several times, followed by drying in a vacuum oven at 60 °C for 12 h.

S2. Chemical structure and components of the samples

The FT-IR spectra of GO, pristine PANI, MOS_2/rGO, and MOS_2-PANI/rGO nanocomposites are illustrated in **Fig. S1**. The characteristic absorption bands at ~3420 and ~2920 cm^{-1} represented -NH_2/-OH, and -CH_2/-CH_3 groups. The characteristic peaks ascribed to aromatic C=C (~1627 cm^{-1}) and C–O groups (C–OH at ~1403 cm^{-1} and C–O at ~1086 cm^{-1}) are observed in the FTIR spectra of GO and MOS_2/rGO, and MOS_2-PANI/rGO samples. The intensity of absorption band at 1730 cm^{-1} for carboxylate C=O stretching was weakened in MOS_2/rGO, whereas the peak disappeared in MOS_2-PANI/rGO, indicating the reduction of GO to rGO.[3] The bands at ~1580 cm^{-1} (C=N stretching), ~1495 cm^{-1} (C=C stretching), ~1289 cm^{-1} (C–N vibration),[4] ~1129 cm^{-1} (polaron formation of PANI (Q = N^+H–B or B–N^+H–B)),[5] ~799 $cm^{-1}$and (ring C–C bending vibration) prove the presence of PANI in MOS_2-PANI/rGO nanocomposite.

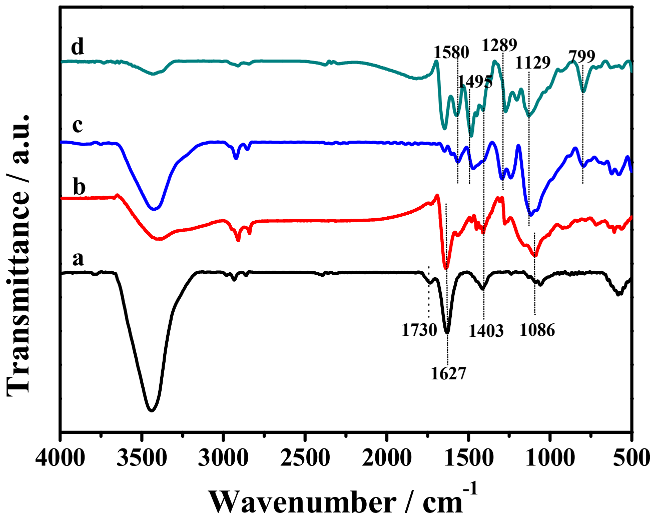

Fig. S1 FT-IR spectra of (a) GO, (b) MoS_2/rGO, (c) PANI and (d) MoS_2-PANI/rGO nanocomposite.

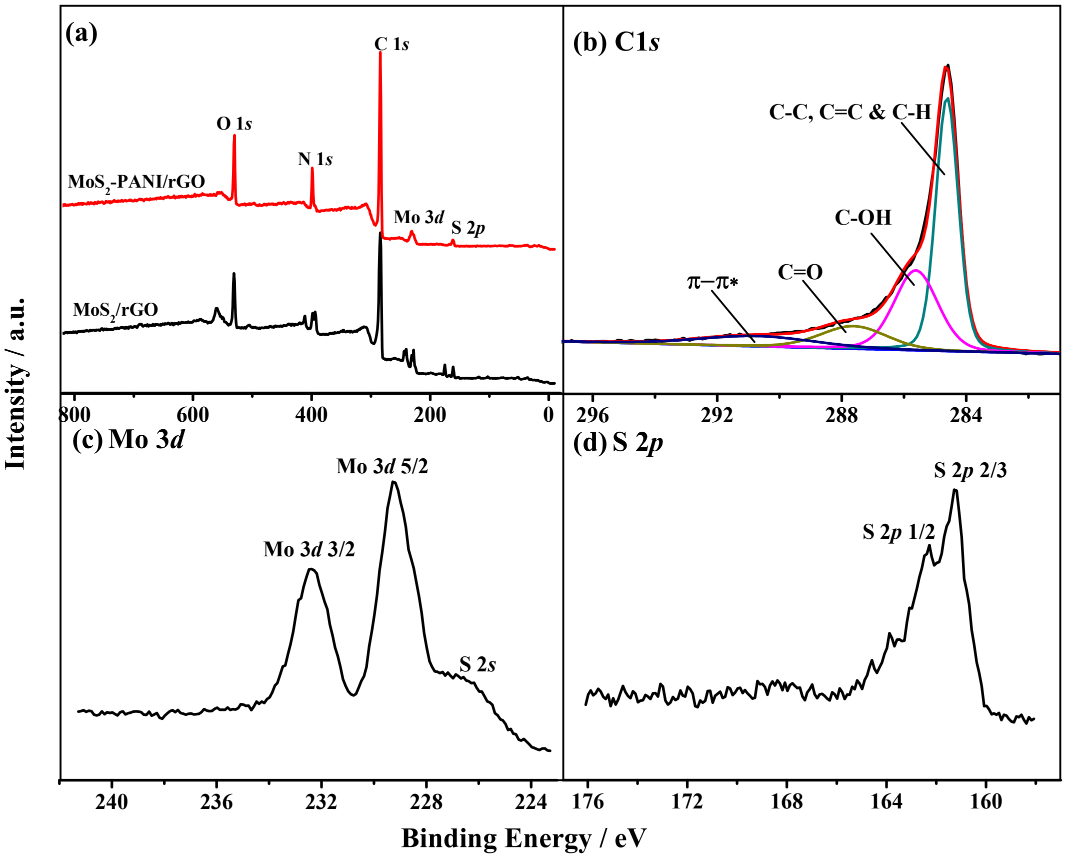

Fig. S2 (a) XPS survey spectra of MoS_2/rGO and MoS_2-PANI/rGO; high-resolution XPS spectra of (b) C 1*s*, (c) Mo 3*d*, and (d) S 2*p* of MoS_2/rGO.

Table S1 The atomic% of C 1*s*, N 1*s*, O 1*s*, Mo 3*d*, and S 2*p* of MoS_2/rGO and MoS_2/rGO/PANI.

**Samples**	**Atomic %**				
	**C 1*s***	**N 1*s***	**O 1*s***	**Mo 3*d***	**S2*p***
MoS_2/rGO	82.9	-	14.7	0.77	1.7
MoS_2-PANI /rGO	74.5	11.8	10.8	0.88	2.0

S3. SEM and TEM images of the samples

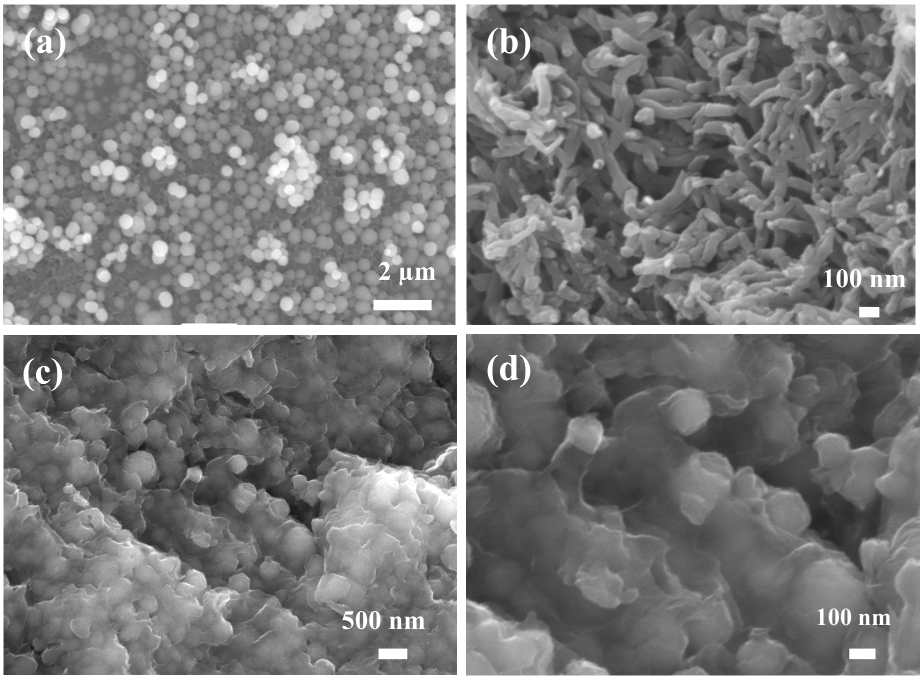

Fig. S3 FE-SEM images of (a) MoS_2 nanospheres, (b) PANI, and (c-d) MoS_2/rGO nanocomposite.

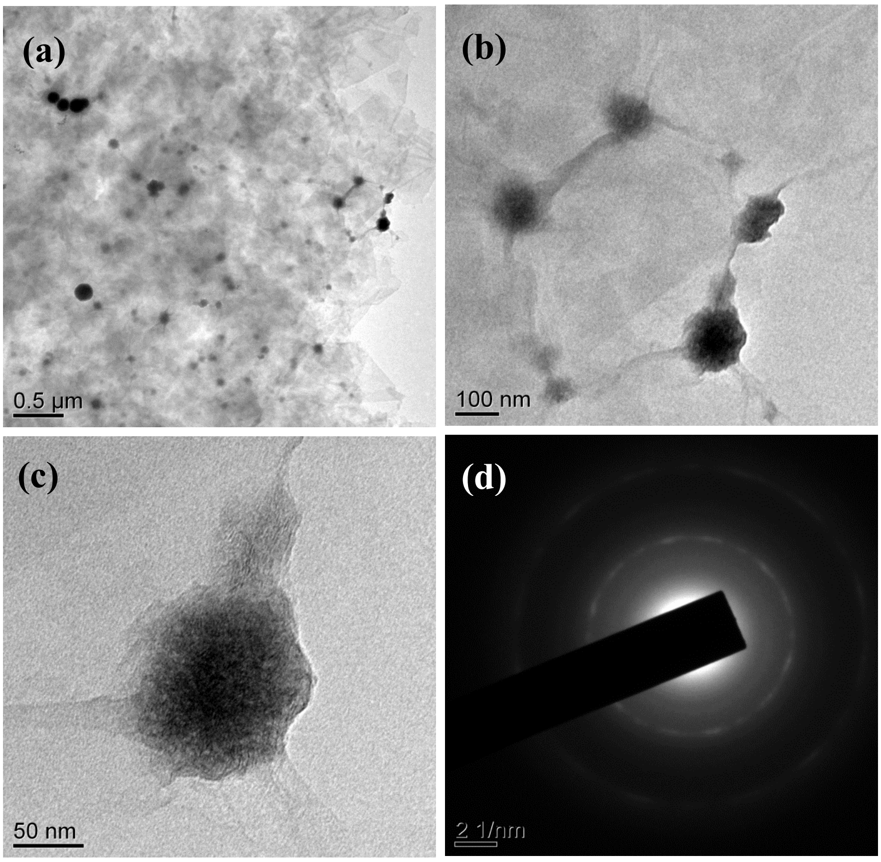

Fig. S4 (a-b) TEM and (c) HR-TEM images of the MoS_2/rGO, and (d) the corresponding SAED pattern.

S4. Electrochemical active surface area of the as-synthesized nanomaterials

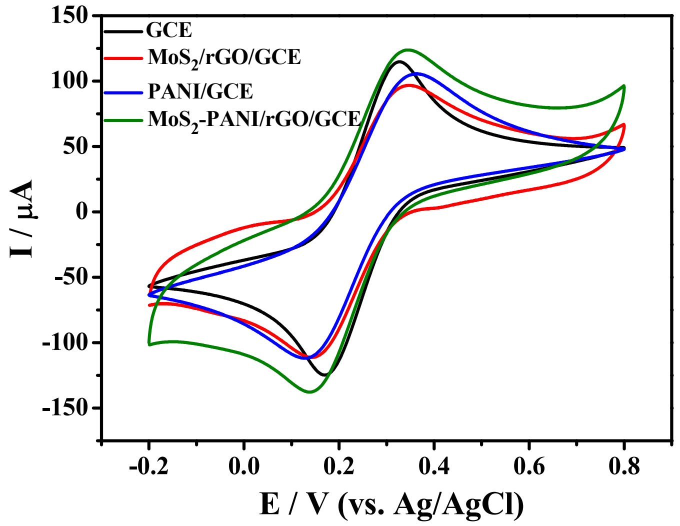

Fig. S5 CV curves of the bare GCE, MoS_2/rGO/GCE, PANI/GCE, and MoS_2-PANI/rGO/GCE at scan rate of 100 $mV{\cdot}s^{-1}$ in 0.1 M PBS (pH 7.0) containing 5 mM $[Fe(CN)_6]^{3-/4-}$ and 0.1 M KCl.

CV measurements were used to evaluate the electrochemical performance of different materials modified electrodes. **Fig. S5** showed the CV curves of bare GCE and MoS_2/rGO, PANI, MoS_2-PANI/rGO-modified GCEs recorded in 0.1 M PBS (pH 7.0) containing 5 mM $[Fe(CN)_6]^{3-/4-}$ and 0.1 M KCl at a scan rate of 100 $mV{\cdot}s^{-1}$. Compared with the bare GCE, the MoS_2/rGO, PANI-modified electrode showed weak redox peaks, suggesting the film acted as a barrier for the electron transfer. For the MoS_2-PANI/rGO-modified GCE, the peaks became more significant, indicating that the MoS_2-PANI/rGO can enhance the electron transfer at the electrolyte/electrode interface. The electrochemical active surface area (A) of different electrodes was estimated according to the Randles–Sevcik equation [6] as follows:

$I_p = 2.69\times10^5\ (n^{3/2})AD^{1/2}v^{1/2}C$

wherein for $Fe(CN)_6^{3-}$, n=1, D = 6.7 $\times10^{-6}$ cm $^{2}\cdot s^{-1}$, C is the concentration of the probe molecule in the solution (mol·cm^{-3}), I_p equals I_{pc}, and v represents the scan rate of CV test. The calculated A values for the bare GCE and MoS_2/rGO, PANI, MoS_2-PANI/rGO-modified GCEs were 0.113, 0.101, 0.102, and 0.125 cm^2, respectively. Therefore, the MoS_2-PANI/rGO/GCE showed the largest electroactive surface area in the different electrodes.

S5. Reproducibility of the developed biosensor

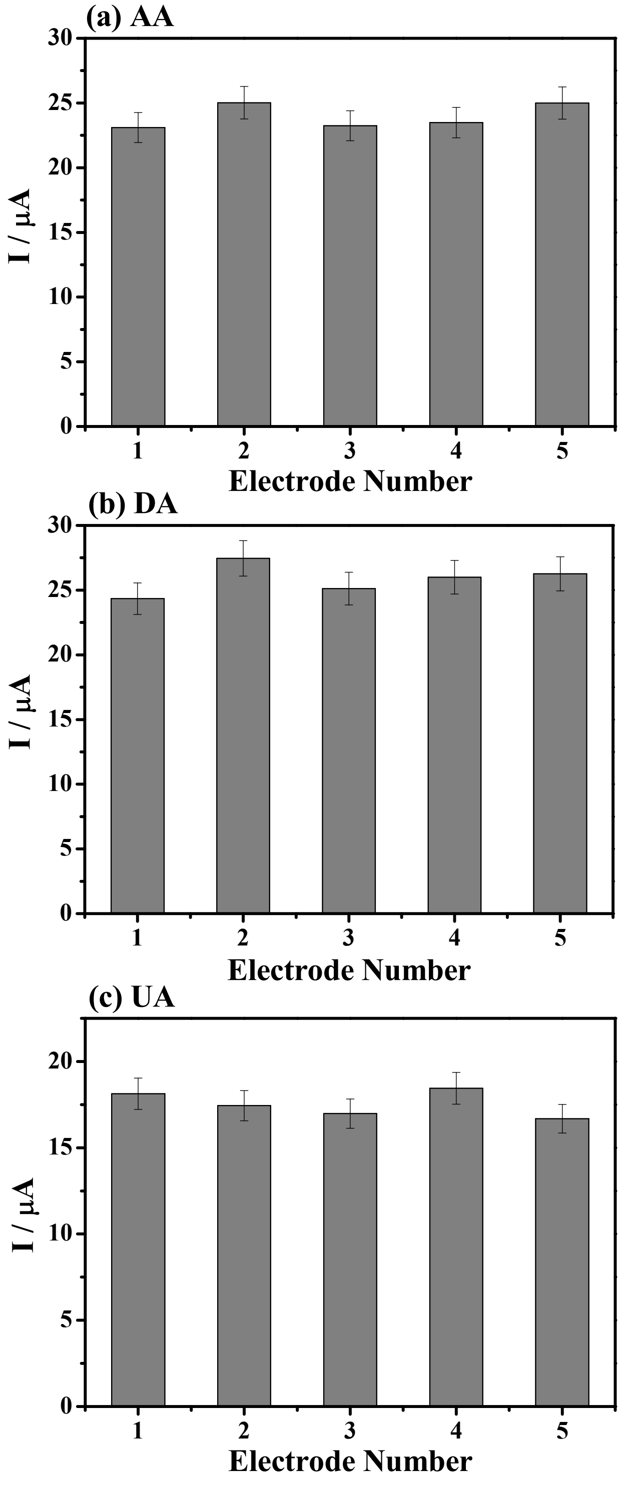

Fig. S6 Peak currents of five MoS_2/rGO modified Au electrodes in 0.1 M PBS (pH 7.0, 0.1 M KCl) containing 1.0 mM, 75 μM DA, and 75 μM UA for detection of (a) AA, (b) DA, and (c) UA.

S6. Real sample analysis

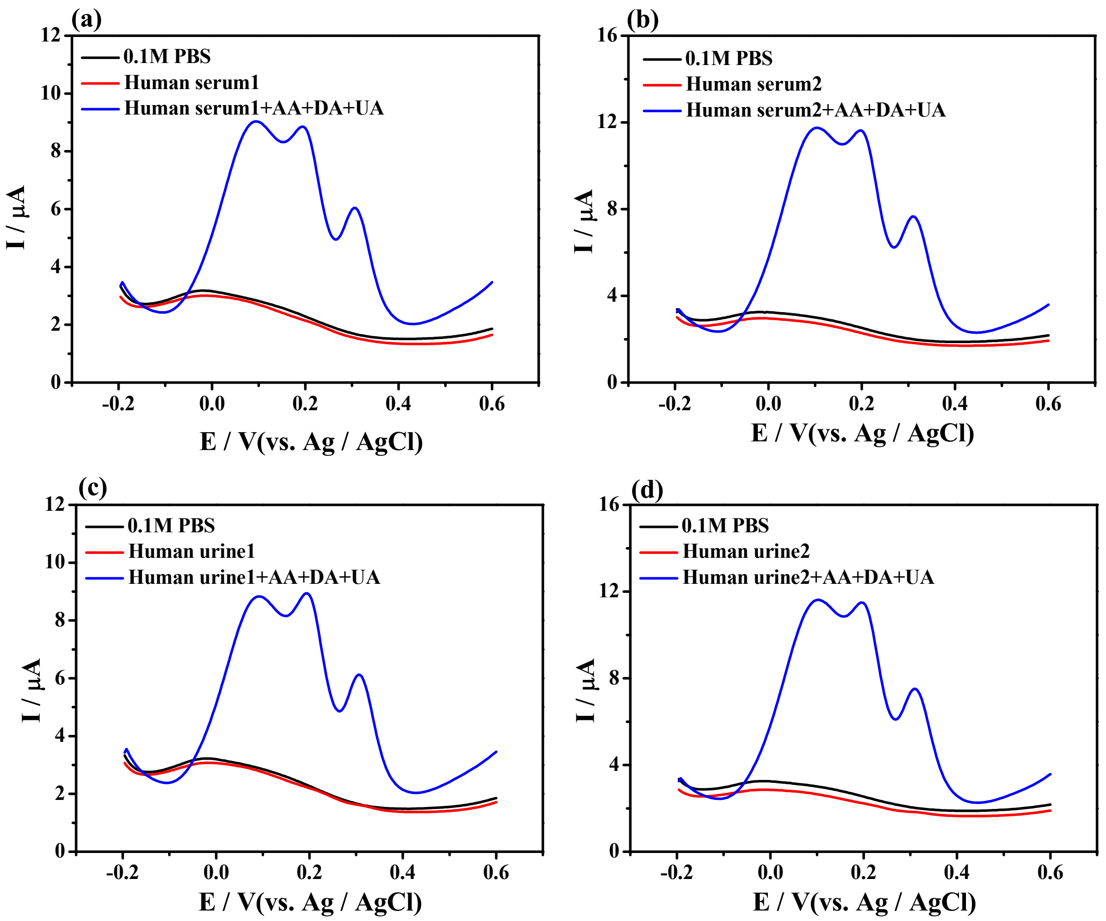

Fig. S7 DPV curves of MoS_2-PANI/rGO/GCE in real samples before and after spiking with AA, DA, and UA: (a) human serum[1], (b) human serum[2], (c) human urine[1], and (d) human urine[2].

References

1. W. S. Hummers Jr and R. E. Offeman, *J. Am. Chem. Soc.,* 1958, **80**, 1339-1339.
2. Z. Zhang, F. Duan, L. He, D. Peng, F. Yan, M. Wang, W. Zong and C. Jia, *Microchim. Acta*, 2016, **183**, 1089-1097.
3. Y. Ren, J. Zhang, Y. Liu, H. Li, H. Wei, B. Li and X. Wang, *ACS Appl. Mater. Inter.*, 2012, **4**, 4776-4780.
4. Y. Zhu, E. Liu, Z. Luo, T. Hu, T. Liu, Z. Li and Q. Zhao, *Electrochim. Acta*, 2014, **118**, 106-111.
5. A. Garai and A. K. Nandi, *Synthetic Met.*, 2009, **159**, 1710-1716.
6. J. Lu, I. Do, L. T. Drzal, R. M. Worden and I. Lee, *ACS Nano*, 2008, **2**, 1825-1832.
